# Numerical study of high‐intensity focused ultrasound (HIFU) in fat reduction

**DOI:** 10.1111/srt.13280

**Published:** 2023-01-13

**Authors:** Sare Mortazavi, Manijhe Mokhtari‐Dizaji

**Affiliations:** ^1^ Department of Medical Physics, Faculty of Medical Sciences Tarbiat Modares University Tehran Iran

**Keywords:** acoustical pressure, fat, high‐intensity focused ultrasound, temperature distribution

## Abstract

**Introduction:**

This study aimed to investigate the effect of fat‐layer thickness and focal depth on the pressure and temperature distribution of tissue.

**Methods:**

Computer simulations were performed for the skin–fat layer models during high‐intensity focused ultrasound (HIFU) treatment. The acoustic pressure field was calculated using the nonlinear Westervelt equation and coupled with the Pennes bioheat transfer equation to obtain the temperature distribution. To investigate the effect of the thickness of the fat layer on pressure and thermal distributions, the thickness of the fat layer behind the focal point (*z* = 13.5 mm) changed from 8 to 24 mm by 2 mm step. The pressure and temperature distribution spectra were extracted.

**Results:**

The simulated results were validated using the experimental results with a 98% correlation coefficient (*p* < 0.05). There was a significant difference between the pressure amplitude and temperature distribution for the 8–14 mm thickness of the fat layer (*p* < 0.05). By changing the focal point from 11.5 to 13.5 mm, the maximum acoustic pressure at the focal point increased 66%, and the maximum temperature was 56%, respectively.

**Conclusion:**

Considering the specific treatment plan for each patient, according to the skin and fat layer thicknesses, can help prevent side effects and optimize the treatment process of HIFU.

## 1 INTRODUCTION

For many years, liposuction was the only treatment for body contouring, which, like other surgical procedures, is not without its complications. As a result, the number of noninvasive methods for removing unwanted fat has increased significantly. There are several methods using energy‐concentrated devices, such as lasers,^1^ radiofrequency,^2^, ^3^ cryolipolysis,^4^, ^5^ ultrasound,^6^ and injection lipolysis,^7^ which can damage adipose tissue through apoptosis or necrosis of adipose cells. Among the noninvasive methods, high‐intensity focused ultrasound (HIFU) is the most powerful therapeutic device that can deliver brilliant results during treatment. HIFU can take energy to the desired depth and remove fat cells without damaging adjacent tissues.^8^, ^9^, ^10^, ^11^, ^12^

HIFU increased the target tissue temperature rapidly to 56–90°C and causes severe necrosis in the focal area. The advantage of this method over other methods is the minimal amount of damage to adjacent tissues and the concentration of heat in the focal area as well as the minimum recovery time. An imaging system could be combined with therapy, and this combination technology has made coagulation procedures more reliable and practical. Minor inflammation and bruising due to the mismatch of ultrasonic waves between the transducer and the skin are among the limitations of this method. Various fat‐loss devices have been suggested, some of which like LipoSonix have been approved by the FDA.^13^ There are two mechanisms for HIFU waves that lead to the destruction of adipose tissue. One is the mechanical effects that immediately disrupt cell membranes. The mechanical ultrasound wave travels through the adipocytes, creating cycles of increased and reduced pressure, which draw gas out of the solution in the form of bubbles. When these bubbles implode, they release energy, causing further mechanical damage to the targeted adipocytes.^10^, ^11^ In addition, the other is heat that destroys excess fat cells.^14^, ^15^, ^16^ In this study, the thermal aspect of HIFU is studied.

HIFU causes rapid heating to temperatures exceeding the upper limit of protein denaturation, resulting in coagulative necrosis. As a result of heat, the temperature at the focal point reaches above 58°C, leading to immediate cell death in the target area. This destruction occurs when adjacent tissues remain intact. Following HIFU treatment, dead cells induce wound healing and invasion of macrophages and other cells, and lipid uptake and transfer from remote areas. Most fat cell destruction occurs within the first 12 weeks after treatment and 95% localized fat cell destruction within the first 18 weeks after treatment. These changes occur without a significant increase in blood plasma lipids. The large flood of cells to heal the wound will absorb the inflammatory cells and then increase the fibroblasts. This process, by heat‐denaturing collagen, generates new collagen along with skin lift.^8^, ^9^, ^17^ HIFU is a method of noninvasive tissue heating and ablation currently used for treating a variety of disorders, including shock wave lithotripsy, uterine fibroids, and solid tumors (prostate cancer, breast cancer, pancreas, tumors Brain, etc.) has been considered.^18^

Due to the widespread use of HIFU transducers in cancer treatments, numerous numerical studies have been conducted to optimize the process of HIFU treatment and to investigate the effective parameters in HIFU thermal distributions.^19^, ^20^, ^21^ But numerical studies have not been reported for cosmetic HIFU treatment, including rejuvenation and fat reduction. In this study, due to the high nonlinear coefficient of adipose tissue and the nonlinear propagation of ultrasonic waves, by increasing the thickness of the subcutaneous fat layer, the pressure and temperature distribution in the focal area was calculated. For calculated acoustic pressure, the Westervelt equation was used. Using the Pennes bioheat transfer equation (BHTE) temperature distribution was obtained at any point. The effect of the thickness of the patient's fat layer on the selection of the focal depth and the ultrasonic transducer with the appropriate focal length was investigated. In this study, the need for a specific treatment plan for each patient according to the thickness of the fat layer was emphasized. Choosing the appropriate physical parameters of the transducer, such as input acoustic intensity, probe cross section, and sonication time for choosing optimal focal length to reduce treatment time is essential.

## 2 MATERIALS AND METHODS

The simulation was performed using the Multiphysics Simulation software (COMSOL; V. 5.3, COMSOL Co., Stockholm, Sweden).

### 2.1 Physics theory

In this study, an acoustic module was used to calculate acoustic pressure. To obtain the acoustic pressure, the nonlinear propagation equation (Westervelt) of the ultrasonic waves is used. The following full‐wave Westervelt equation, which is based on the effects of diffraction, absorption, and nonlinear propagation, is used^19^, ^20^, ^21^, ^22^, ^23^, ^24^, ^25^:

(1) $$\nabla^{2}p-\frac{1}{c^{2}}\frac{\partial^{2}p}{\partial t^{2}}+\frac{\delta }{c^{2}}\frac{\partial }{\partial t}\left(\nabla^{2}p\right)=\frac{\beta }{\rho_{0}c^{4}}\frac{\partial^{2}}{\partial t^{2}}\left(p^{2}\right)\;$$

(2) $$\delta =\frac{1}{\rho_{0}}\left(\frac{4}{3}\mu +\mu_{B}+\frac{\left(\gamma -1\right)k}{c_{p}}\right)$$

where *p*, *c*, *ρ*, and *δ* are the acoustic pressure, speed of the sound propagation in the medium, density, and the diffusivity of sound (Equation 2), respectively, and *β* $\left(1+\frac{B}{2A}\right)$ is a nonlinearity coefficient. In Equation (2), *μ*, *μ_B_*, *γ*, *k*, and *c_p_* are the dynamic viscosity coefficient, the bulk viscosity coefficient, the heat capacity ratio, the thermal conductivity, and the specific heat capacity, respectively. In Equation (1), the first two statements show the Westervelt equation for linear behavior, the third statement shows the loss of thermal conductivity and fluid thermo‐viscous property, and the last term refers to nonlinear wave behavior with finite amplitude. To calculate the temperature, the following Pennes BHTE is used^25^, ^26^, ^27^:

(3) $$\rho_{t}c_{t}\frac{\partial T_{t}}{\partial t}=\;k_{t\;}\nabla^{2}T_{t}-\;w_{b}c_{b}\left(T-T_{b}\right)+\;Q\;$$

where indices *t* and *b* are tissue and blood parameters, respectively. w_*b*_ and *Q* are the blood perfusion rate and external heat source, respectively. *Q* represents the volumetric heat generation due to the absorption of acoustic intensity in the tissue domain and can be calculated by the following equation:

(4) $$Q\;=\;2\alpha \frac{P_{rms}^{2}}{\rho c}$$

(5) $$p_{rms}^{2}=\frac{1}{\;t_{f}-t_{i}}\int_{t_{i}}^{t_{f}}p^{2}dt\;$$

where *α* and $P_{rms}$ are the absorption coefficient of tissue and root mean square (rms) of acoustical pressure.^27^, ^28^ *t_i_* and *t_f_* are the start and end times of integration. This interval should be in the part of the solution, in which the pressure behaves in an orderly manner. After performing the calculations, energy is obtained at each point. To calculate the pressure field using compressive acoustic physics, the pressure distribution in the adipose tissue as the target tissue using the full‐wave nonlinear Westervelt equation (Equation 1) is obtained. Pressure contours are plotted in axes *z* (distance from the surface transducer) and *r* (radial direction). In order to determine temperature distribution, the solution of the full‐wave Westervelt Equation (Equation 1) is coupled with the Pennes BHTE (Equation 3), as the external heat source in the bioheat equation. According to Equation (4), the instantaneous field is obtained in any region. Temperature contours are plotted on the plane (*z*–*r*).

### 2.2 Model geometry

**FIGURE 1:**
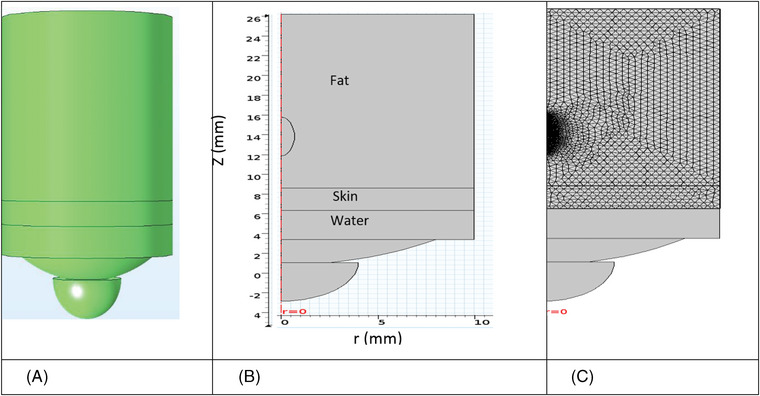
Two‐dimensional view of the model for 18 mm thickness of the fat layer. The longitudinal and transverse axes are in millimeters: (A) 3D, (B) *z*–*r* plane, and (C) thermal meshing

The spherical transducer of 4 MHz frequency, 13.5 mm curvature radius, 8 mm radius, and 4 mm hole radius was simulated (according to Liposonix, Solta Medical Inc., Hayward, CA, USA). A two‐dimensional view of the model, including the three layers of water, skin, and fat, with axial symmetry, is shown in Figure 1. The geometric dimensions of the cylindrical model are 16–32 mm long (for the thickness of the fat layer from 8 to 24 mm) and 10 mm wide.

The physical properties of each of the two layers of skin and fat for simulation are given in Table 1. The distance between the transducer and the tissue is filled with water (due to the acoustic impedance matching).

**TABLE 1:** Physical properties of the two layers of skin, fat, and also, water as matching layers^29^, ^30^, ^31^, ^32^, ^33^

Fat	Skin	Water	Parameter
1450	1640	1430	Speed of sound (m/s)
0.18	0.44	0.6	Thermal conductivity (W/mC)
2674	3300	4200	Heat capacity (J/kg^3^)
911	1100	1000	Density (kg/m^3^)
6.5	4.9	3.5	Nonlinear coefficient
0.60	1.80	0.01	Attenuation coefficient at 1 MHz (dB/cm)
0.04	0.024	0.024	Bulk viscosity (Pa s)
0.3	0.008	0.008	Dynamic viscosity (Pa s)
8–24	2.35	3.0	Thickness (mm)

Two sets of boundary conditions are needed for simulation wave propagation in a two‐dimensional space. The first boundary condition is used for solving the acoustic equation and the second boundary condition for solving the heat transfer equation^22^, ^34^, ^35^: The sound pressure equation of the source is assumed as *p* = *p*_0_sin (*ωt*), and it is assumed that reflection and transmission coefficients are zero (absorbed by the medium):

(6) $$-\vec{n}\left(-\frac{1}{\rho }\nabla p\right)=\frac{1}{Z_{mat}}\frac{\partial p}{\partial t}\;$$

where Z*_mat_* is the medium impedance, in which the boundary condition is imposed on the boundary of that medium. $\vec{n}$ is a normal vector of a surface. The initial conditions for the acoustic equations are *p* = 0 and $\frac{\partial p}{\partial t}$ = 0, and the initial condition for solving the heat transfer is defined as *T* = *T*_0_ = 310 K.

### 2.3 Meshing

Mesh size, in which the equation is solved, should be small enough to take into account the peak and subdivisions of the wave to accurately calculate the propagation of a wave during the medium.

Therefore, an ultrasonic transducer has to be divided into several elements. The longer the transducer area is divided into more elements, the better the wavelength resolution would be. Here, the elements are considered to be smaller than one fourth of the wavelength (*λ*/4), and the focal size is also considered to be *λ*/8. In calculating the pressure field, the time step is of order 10^−9^ s, and the time of solving the transient equation is of order 9 × 10^−6^ s. For the temperature field, the solution time is considered to be of order 10^−3^ s. The difference in the two‐time scales used is attributed to multi‐physical numerical modeling, in which the acoustic wave takes about 1 μs (50–60 μs in this study) to reach the desired region.^21^, ^22^, ^35^, ^36^, ^37^ Thermal meshing is shown in Figure 1C.

The numerical solution method in this study was validated using experimental results obtained from Kim et al.^38^ An HIFU probe was placed in a chamber filled with water (18°C) that can move by a stepping motor. The irradiation time was less than 40 ms with a 3 s total time. The probe stops at given intervals and emits a 4 MHz focused ultrasound and a 9 mm focal length. The tissue‐mimicking phantom was fabricated using 10% carrageenan gel, which has ultrasonic characteristics similar to human tissue. The temperature distribution in the phantom was observed using thermochromic films.

In the experimental study conditions (9 mm focal length, 21 W input power, 4 MHz frequency, and the same phantom's physical parameter), modeling is done. The results of temperature changes in terms of the depth of the simulation and experimental data were analyzed by Pearson's correlation analysis and estimated at a 95% confidence level (*p* < 0.05).

## 3 RESULTS

### 3.1 Validation of model

**FIGURE 2:**
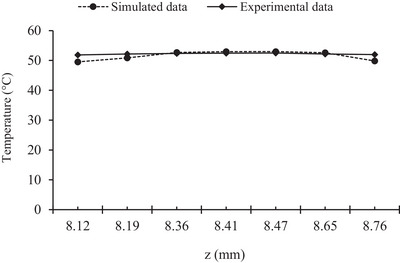
Comparing temperature changes in the focal area using two methods of numerical simulation and experimental results

At the same focal depth, there was a significant correlation between the temperature changes in the simulated method and experimental results (*R* = 0.98, *p* < 0.05). The validation results demonstrated a reasonably good agreement between profiles obtained from results present in numerical simulation and those reported in Ref. [38] with a 95% confidence level (Figure 2).

Because of the limitation of the experimental data and long simulation time in the time domain, the temperature changes were simulated for 0–14 mm depth, and the temperature values at the focal depth were compared. The input sound intensity was selected at 30 W/cm^2^. The radius of the transducer was selected according to the thickness of the fat layer with 8 mm penetration depth and 13.5 mm radius of curvature (ROC) and 16 mm aperture diameter. These characterizations with a penetration depth of 13 mm are widely used in the treatment of body contouring in medical clinics.^39^ The thickness of the layers is selected as 3.00 mm water layer, 2.35 mm skin layer,^29^ and 8.00, 10.00, 12.00, 14.00, 16.00, 18.00, 20.00, 24.00 mm fat layer. The *λ*/4 spatial resolution is equal to 9 × 10^−5^ m, and the time step is in the range 0 and the final time according to the relation *l*/*c* for each length of the geometry (varies from 9 to 25 μs depending on the thickness of 8–24 mm). Each diagram was drawn at the last time of calculating the pressure field.

### 3.2 The pressure nonlinear model

**FIGURE 3:**
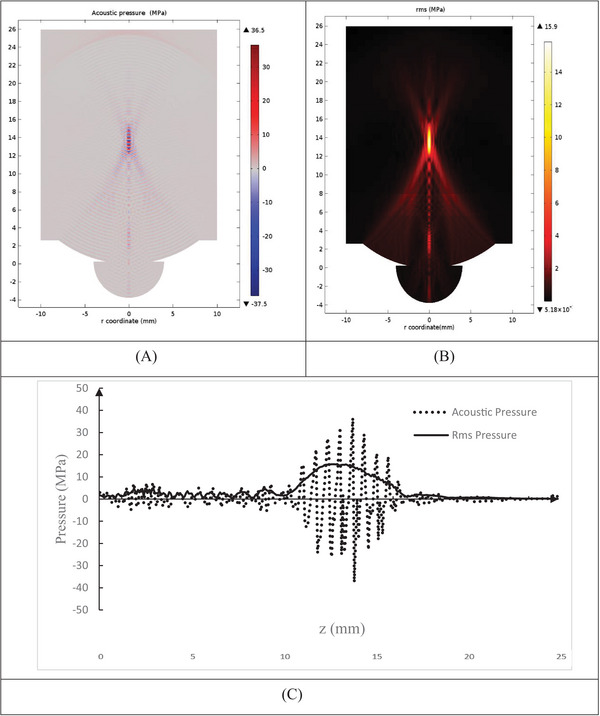
Pressure (MPa) contours on (*z*–*r*) plane (mm): (A) acoustic pressure (MPa), (B) root mean square (rms) pressure (MPa), for 18 mm thickness of the fat layer, and (C) rms pressure distributions (MPa) in axial (*z*) direction (mm)

The pressure equations in the nonlinear model were applied using the Westervelt equation (Equation 1) and the Pennes BHTE (Equation 3). In calculating the temperature field, rms pressure was used. The Westervelt equation was calculated to solve the acoustic pressure field in the same physical condition for different thicknesses of the fat layer (8–24 mm, step interval 2 mm). Acoustical pressure (MPa) and rms pressure (MPa) contours on (*r*–*z*) plan and axial direction (*z*) (mm) for a thickness of 18 mm of a fat layer are simulated in Figure 3.

Maximum acoustic pressure on the (*z*–*r*) plane at the focal point (*z* = 13.5 mm) reached 36.5 MPa, and maximum rms pressure at the focal point (*z* = 13.5 mm) reached 15.9 MPa (Figure 3). The distribution of acoustic pressure, due to its instantaneous nature and time dependence, has severe fluctuations in the focal region and is highly dependent on the calculated time step and final time. Due to the difference in depth of the model in different thicknesses of the fat layer, the final calculation time of the pressure field has been different and may lose parts of the existing peaks. rms pressure smoothed out momentary pressure fluctuations at the center by averaging the momentary moment of pressure according to (Equation 5) Figure 3C. Due to the model validation, in this study, pressure (rms) is used as the reference pressure in calculating the temperature distribution.

**FIGURE 4:**
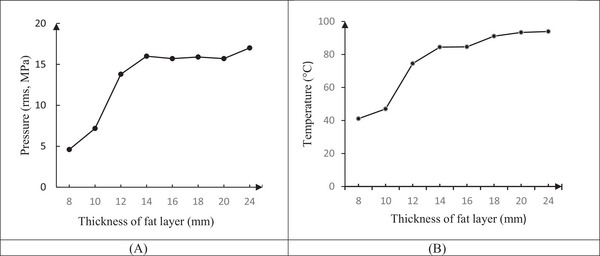
(A) Maximum pressure (rms, MPa) diagram and (B) maximum temperature, at focal point (*z* = 13.5 mm) for thicknesses 8, 10, 12, 14, 16, 18, 20, and 24 mm of fat layer

Maximum pressure (rms, MPa) at focal point (*z* = 13.5 mm) for thicknesses 8, 10, 12, 14, 16, 18, 20, and 24 mm of fat layer is shown in Figure 4A. With an increasing thickness of the fat layer (8–24 mm), the maximum pressure (rms) increased (from 4.6 to 16 MPa), in constant physical parameters. According to the results, increasing the thickness of the fat layers from 8 to 14 mm due to their proximity to the focal area, there is a significantly different in maximum pressure (*p* < 0.05). However, at 16–24 mm thicknesses, with increasing distance from the focal area, increasing the thickness of the fat layer did not affect the maximum temperature at the focal point (*p* < 0.05) (Figure 4B).

**FIGURE 5:**
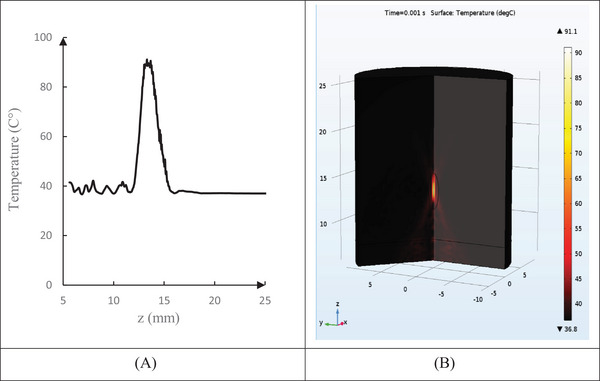
3D temperature (°C) distribution for 18 mm thickness fat layer: (A) temperature distribution in axial (*z*) direction (mm) and (B) 3D temperature contour

After calculating the rms of pressure and placing it in Equations (3) and (4), the temperature distribution is estimated. The three‐dimensional shape of the temperature field (°C) at the same time 1 ms for thicknesses of 8, 10, 12, 14, 16, 18, 20, and 24 mm fat layer was obtained. For example, for the 18 mm thickness of the fat, a layer is shown in Figure 5. The maximum temperature occurred at the focal point (*z* = 13.5 mm). For the 18 mm fat layer thickness, the maximum temperature reached 91.1°C at the focal point (Figure 5A). To compare the temperature behavior at different thicknesses, the same sonication time is considered 0.001 s. But it is obvious in clinical, the sonication time is reduced to the desired temperature of the device (60–70°C).

In the maximum temperature at the focal point (*z* = 13.5 mm) for 8, 10, 12, 14, 16, 18, 20, and 24 mm, the fat layer thicknesses is shown in Figure 5A with 1 ms sonication time. With increasing the fat layer thickness, the maximum temperature at the focal point increases. This trend of increasing from 8 to 14 mm thickness of the fat layer was a sharp edge and from the thickness of 14 mm onward due to distance from the focal point, increasing the thickness of the fat layer does not affect the maximum temperature at the focal point (*z* = 13.5 mm).

Maximum pressure (MPa) and temperature (°C) at the focal point (*z* = 13.5 mm) for fat layer thicknesses 8, 10, 12, 14, 16, 18, 20, and 24 mm are shown in Table 2.

**TABLE 2:** Maximum pressure (rms, MPa) and temperature (°C) at the focal point (*z* = 13.5 mm) for fat layer thickness

Fat layer (mm)	Maximum pressure (MPa)	Maximum temperature (°C)
8	4.1	41.1
10	7.1	47.7
12	13.8	74.5
14	15.6	84.4
16	15.7	84.6
18	15.9	91.1
20	16.0	93.4
24	16.1	93.9

### 3.3 Increasing the fat thickness

**FIGURE 6:**
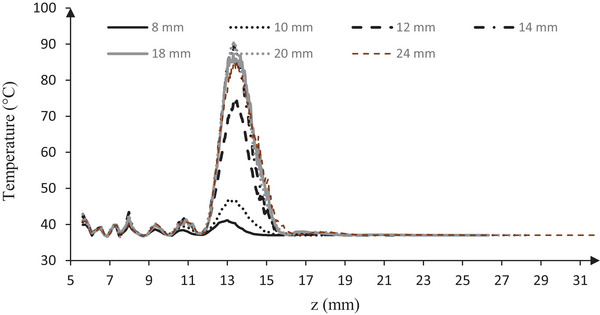
Temperature (°C) range in axial (*z*) direction (mm) for 8, 10, 12, 14, 16, 18, 20, and 24 mm fat layer

To better understand the distribution of temperature by increasing the thickness of the fat layers, the temperature range in terms of depth for each model from the beginning of skin tissue (*z* = 5.1–7.4 mm) to the end of the fat layer (*z* = 7.4–25.3 mm) was extracted separately (Figure 6). The temperature distribution in the axial direction (*z*) is shown. The temperature varies in thickness of 8, 10, and 12 mm (Figure 6). With increasing thickness due to the distance from the focal point, the spectra are almost on top of each other. It should be noted that except for the focal range (from 12 to 15 mm), in other places, such as the skin layer (from 5.1 to 7.4 mm) and the pre‐focal fat layer (from 7.4 to 12.0 mm). Moreover, the after focal length (15.0–32.0 mm), the temperature range for variable thicknesses is the same.

The maximum pressure and temperature occurred at the focal point. An increase in fat thickness has no effect on the focal point location (∼4 mm).

The effect of increasing the fat thickness on the temperature‐induced in the nonlinear model was analyzed by ANOVA. There is a significant difference between temperatures at 8, 10, 12, and 14 mm fat thicknesses (*p* < 0.05). However, there is no significant difference between temperatures from a thickness of 16 onward (*p* > 0.05).

### 3.4 Pressure and temperature distributions with changing the focal length

**FIGURE 7:**
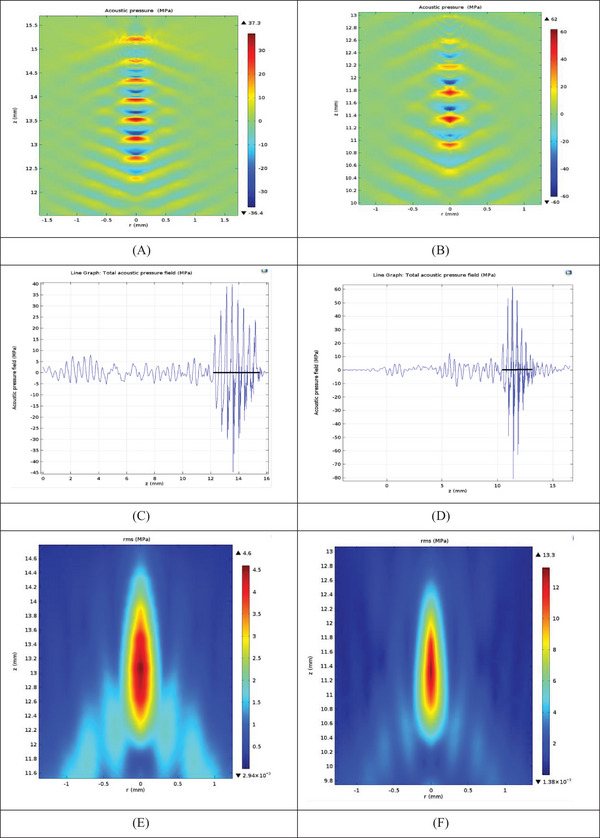
Acoustic pressure (MPa) contour in (*r*–*z*) plane (mm) with a radius of the curvature of (A) 13.5 mm and (B) 11.5 mm; acoustic pressure (MPa) in axial direction (*z*) (mm) with a radius of the curvature of (C) 13.5 mm and (D) 11.5 mm; root mean square of pressure (MPa) contour in (*r*–*z*) plane (mm) with a radius of the curvature of (E) 13.5 mm and (F) 11.5 mm

At constant physical conditions (30 W/cm^2^ intensity, 16 mm aperture diameter) and 8 mm fat thickness, the focal length was changed. By changing the focal length from 13.5 to 11.5 mm, the acoustic pressure (MPa) contours, rms pressure (MPa) on (*z*–*r*) plane, are plotted in Figure 7.

With the decreasing focal length of 13.5–11.5 mm and 8 mm fat thickness, the maximum acoustic pressure at the focal point increased from 37 to 62 MPa (Figure 7A,B). The thermal necrosis in the axial direction (*z*) from 3.1 to 1.7 mm decreased (Figure 7C,D). The rms of pressure increased from 4.6 to 13.3 MPa (Figure 7E,F).

**FIGURE 8:**
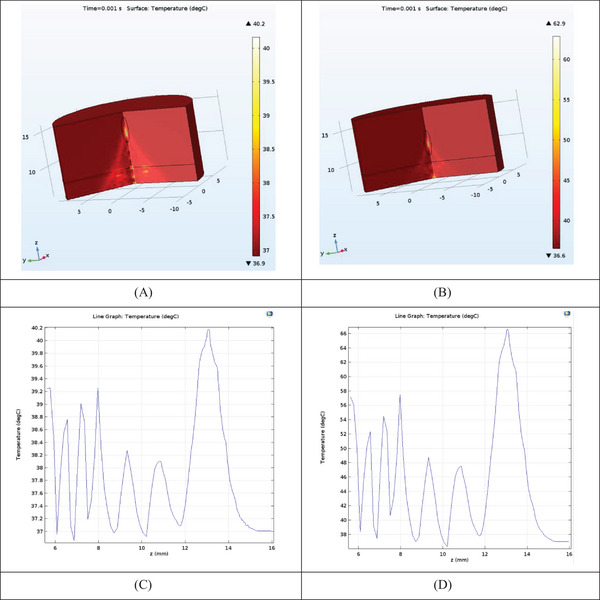
3D temperature contour (°C) in *z*–*r* plane (mm) with a radius of curvature: (A) 13.5 mm and (B) 11.5 mm; temperature distribution (°C) in axial (*z*) direction (mm) with a radius of curvature: (C) 13.5 mm and (D) 11.5 mm

After calculating temperature according to Equations (3) and (4), 3D temperature (°C) contour at 1 ms sonication time (for ROC = 11.5, 13.5 mm) is plotted in Figure 8. With decreasing focal length and the ROC from 13.5 to 11.5 mm (8 mm fat thickness), the maximum temperature at the focal point has increased from 40.2 to 62.9°C (Figure 8A,B). The temperature on the skin has also risen (from 39.2 to 57.0°C) (Figure 8C,D). This result emphasizes the need to be careful in choosing the type of treatment plan and the focal length to prevent skin burns.

## 4 DISCUSSION

An HIFU device received Food and Drug Administration clearance for use in a noninvasive brow lift and neck lift in 2009. HIFU has been the representative noninvasive method for skin rejuvenation. Moreover, HIFU was FDA cleared in 2011 for reducing waist circumference.^40^ There are many noninvasive options for body sculpting, such as radiofrequency ablation, cryolipolysis, injection lipolysis, external low‐level lasers, laser ablation, nonthermal ultrasound, and HIFU. Each of these treatments has no admission for treatment without anesthesia or analgesia and typically has fewer complications than liposuction. However, with the exception of HIFU, patients have to visit the hospital several times to completion treatment. Injection lipolysis and cryolipolysis have significant potential for AEs; in contrast, previous clinical studies that supported thermal HIFU for body sculpting have had no serious AEs, including alterations in lipid profiles or other laboratory parameters.^41^ Therefore, many clinicians are keeping an eye on the HIFU technique for the purpose of body sculpting. In addition to local adipocyte necrosis, evidence of collagen remodeling from the thermal effects of HIFU has been observed. Ko et al.^42^ observed significant improvements in two body regions (abdomen and thighs) when targeted for HIFU treatment.

Moreover, no thermal damage on the skin surface of the HIFU treatment site was not observed. Kwon et al.^39^ reported the temperature changes of the porcine model during the HIFU procedure. They showed targeted subcutaneous fat to be around 70°C, whereas the skin surface temperature only went up to 33.1–35.6°C. Therefore, newly developed transducers could effectively and safely deliver HIFU energy deeper into the skin and eventually show body sculpting effects due not only to skin tightening but also to the reduction of subcutaneous fats. A number of articles have reported the safety and efficacy of HIFU over other similar treatments, such as the use of lasers^42^; however, in practice, side effects have been observed after the procedure such as unexpected fat loss, superficial nodules, and burns of underneath the skin. A possible reason for this is the variable thicknesses of the epidermis, dermis, and subcutaneous fat layer according to the anatomical region and patient skin condition.

Park et al.^13^ discussed the impact of treatment parameters on outcomes and side effects. The target depth is defined as 4.5 and 6 mm, which is equal to that of the thermal coagulation point (TCP) depth. Exposures of HIFU were performed at the same power settings (35 W) and 90 ms exposure times. Porcine tissues were examined. They also observed coagulated tissue and measured the width, height, and depth of the TCP. In porcine muscle, TCP was measured 130% deeper compared with the preselected penetration depth. The thermally injured area in the fat layer was approximately six times larger with 4.5 mm HPs compared to the skin exposed to 6.0 mm HPs under the same condition. They evaluated the propriety and accuracy of the new HIFU device as well as the depth of thermal injury created by HIFU according to treatment and skin condition. Therefore, patients and HPs must be chosen carefully with regard to the thickness of the skin and its composition. Their study showed consistent results that changes in the TCP were observed as energy settings were varied. This result indicates that skin thickness and patient condition should be considered during HIFU treatment. The thickness of skin varies among individuals and among regions on the same individual. In conclusion, a holistic consideration of anatomical location underneath the skin as well as skin surface condition should be taken into account for better treatment outcomes of HIFU and for the minimization of unwanted adverse effects, such as subcutaneous fat cell damage, dermal nodules, and serious skin burn.

In this study, in the first part, the effect of increasing the thickness of the fat layer in constant parameters (30 W/cm^2^ input intensity, 4 MHz frequency, and 13 mm focal length) was investigated. With increasing the thickness of the fat layer in the focusing zone (8–14 mm), the maximum temperature increased, and outside this range (14–24 mm), no significant change was observed. In the second part of this study, at the same thickness of the fat layer (8 mm), the focal point was selected 11.5 and 13.5 mm, and simulation was done. By changing the focal length in constant physical conditions to the same thickness as the fat layer, the dimensions of the TCP were investigated. With decreasing penetration depth (focal length from 13.5 to 11.5 mm), the focal area became narrower (almost half), and the temperature increased (56%) at the focal point. Due to the significant effect of fat layer thickness, also the effect of accurate focal point selection on pressure and temperature distribution in modeling, and according to the previous studies,^21^ the effect of the input physical parameters of the transducer on the thermal acoustic design created in the tissue, it is necessary to have a specific treatment plan for each patient according to the skin condition and fat layer thickness.

## 5 CONCLUSION

HIFU is a modern, nonsurgical alternative to operative facial rejuvenation and body contouring. HIFU treatment has shown statistically significant effects on removing unwanted fat and cellulite.

The results in our simulation showed a change in the focal length of the transducer and thickness of a fat layer of the patient; it can be very effective in treating the output. Therefore, it is necessary to provide a treatment plan. Thus, HIFU parameters should be optimized to reduce the treatment time, damage to the surrounding normal structures, and to ensure the safety and efficacy of this modality. Our numerical model can simulate the acoustic and thermal field of HIFU waves with high computational accuracy. Optimization of HIFU treatment planning is feasible to enhance efficacy and safety. Experimental results confirmed this numerical model.

## CONFLICTS OF INTEREST

The authors have no financial conflicts of interest.

## Data Availability

All relevant data are included within the paper and supporting information files. Please contact the corresponding author for material availability.
